# Adrenal lesion classification revisited: validation and adjustment of dual-energy CT derived virtual unenhanced attenuation thresholds

**DOI:** 10.1007/s00261-025-04939-3

**Published:** 2025-04-26

**Authors:** Pascale Bernard, Christian Nelles, Philipp Fervers, Joline Schwan, Kaloyan Dankov, David Maintz, David Zopfs, Nils Große Hokamp, Thorsten Persigehl, Simon Lennartz

**Affiliations:** Institute for Diagnostic and Interventional Radiology, University of Cologne, Faculty of Medicine and University Hospital Cologne, Cologne, Germany

**Keywords:** Multidetector computed tomography, Adrenal glands, Abdomen, Adrenal gland neoplasms

## Abstract

**Objectives:**

Dual-energy CT (DECT)-derived virtual unenhanced (VUE) images have been investigated for adrenal lesion differentiation, yet previously reported thresholds vary, hampering clinical application. We aimed to test previous VUE thresholds for adrenal lesion differentiation in a large retrospective cohort, to provide a cross-validated threshold based on our data, and to investigate the influence of underlying malignancies on differentiation accuracy.

**Methods:**

290 patients with 348 adrenal lesions (169 metastases, 179 adenomas) were included. Dual-layer DECT-derived VUE thresholds from 3 previous studies were retrieved, applied to our cohort and corresponding sensitivity/specificity/accuracy was calculated. Optimal threshold based on our data were determined using ROC-analysis with five-fold cross validation. Moreover, a threshold with similar specificity to the 10 HU threshold in unenhanced images was calculated. Subgroup analysis of adrenal lesion differentiation depending on underlying malignancies was performed.

**Results:**

The previously suggested thresholds were 20, 22 and 29 HU, and corresponding sensitivity/specificity/accuracy was 0.61/0.92/0.76, 0.67/0.91/0.78, and 0.82/0.59/0.71, respectively. The threshold determined from our cohort was 24.7 HU, yielding a sensitivity/specificity/accuracy of 0.76/0.81/0.79. Differentiation in disease-specific subgroups showed similar sensitivity/specificity/accuracy (Melanoma:0.78/0.84/0.79; Lung cancer:0.78/0.8/0.78; RCC:0.78/1/0.79). The VUE threshold to achieve a 0.98 specificity similar to the unenhanced 10 HU cutoff was 17.3 HU, yielding a sensitivity of 0.49.

**Conclusion:**

Previous VUE attenuation thresholds showed a varying accuracy for differentiation between adenomas and metastases. A cross-validated VUE threshold of 24.7 HU yielded a mean accuracy of 0.79, whereas a threshold of 17.3 HU was best for achieving comparable specificity as reported for the 10 HU threshold in unenhanced images.

**Graphical abstract:**

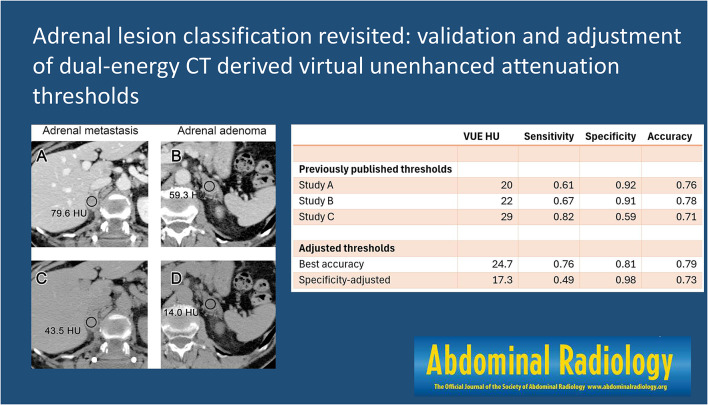

## Introduction

Over the past decades, the number of computed tomography (CT) examinations increased due to technical improvement and better availability [1], which resulted in an increase in incidental findings. Adrenal incidentalomas are one of the most common incidental findings at abdominal CT [2, 3]. For accurate differentiation between benign and malignant adrenal lesions, either multiphase CT or chemical shift MRI are warranted to identify microscopic fat, or a washout of contrast agent [2]. For unenhanced CT, a threshold of 10 HU in unenhanced images allows for diagnosis of 70% of adenomas based on their lipid-rich content [4]. However, most CT protocols used for high-volume indications such as oncologic follow-up are limited to portal venous (PV) phase images, as an additional acquisition of unenhanced images leads to increased radiation exposure for the patient without adding diagnostic value in many instances [5]. Therefore, particularly in baseline staging CT examinations without availability of follow-up, diagnosis of adrenal lesions is often hampered.

Since its clinical introduction, dual-energy CT (DECT) has been investigated for classification of adrenal adenoma using virtual unenhanced (VUE) images [6, 7]. In these images, the iodine content is quantified by means of material decomposition and subsequently subtracted from the grey-scale original image, reflecting the expected attenuation of the corresponding unenhanced image [8, 9]. Throughout the years of DECT research, several different VUE thresholds for adrenal lesion classification have been proposed. However, many of these studies resulted in different VUE attenuation thresholds, were limited regarding their sample size, and did not include internal validation of the suggested cut-offs [10–12]. These limitations hamper clinical application of DECT-derived VUE attenuation thresholds.

In our study, we therefore aimed to test previously published VUE thresholds for adrenal lesion classification derived from a detector-based dual-layer dual-energy CT (dlDECT) and to investigate if an optimized, cross-validated threshold could be suggested. Additionally, we aimed to analyze if the underlying malignancy may influence differentiation of adrenal metastases from adenoma.

## Materials and methods

### Patients

This retrospective study was approved by the institutional review board. The necessity to obtain informed consent was waived due to the retrospective character of the study.

A combined query to the radiological information system (RIS) and picture archiving and communication system (PACS) at our institution was used to identify subjects who underwent an abdominal, PV phase examination of the abdomen and for whom presence of adrenal metastases or adenomas was mentioned in the radiological report. A total of 307 consecutive patients were retrospectively identified, who underwent PV phase abdominal CT on a dlDECT from 15.06.2016 up to 03.12.2021. This inclusion period was chosen to facilitate creating a cohort with a homogeneous scan protocol that had been in effect during that time frame. 17 patients were excluded, 12 due to insufficient or corrupted spectral base image (SBI) data, 4 patients due to lack of eligible lesions according to the reference standard, and one patient due to a lack of PV phase images. Finally, 290 patients were included (mean age 64.6 ± 10.9 years): 171 men (mean age 65.2 ± 10.8 years) and 119 women (mean age 63.8 ± 11.1 years). There were 151 patients with at least one adenoma (125 with one and 26 with two adenomas) and 138 patients with at least one metastasis (112 with one, 25 with two and one with three metastases). Additionally, one patient had two adenomas and one metastasis. This resulted in a total of 348 adrenal lesions, of which 169 were metastases (80 in the right and 89 in the left adrenal gland) and 179 adenomas (69 in the right and 110 in the left adrenal gland). Among the patients with adrenal metastases, 49 had melanoma, 40 lung cancer, 15 RCC and 65 patients were summarized as a subgroup consisting of several different malignancies. The latter group included the following malignancies: transitional cell carcinoma, hepatocellular carcinoma, ovarian cancer, choriocarcinoma, colorectal cancer, gastrointestinal stromal tumor, breast cancer, Merkel-cell carcinoma, esophageal cancer, stomach cancer, prostate cancer, cervical cancer, pancreatic cancer, pulmonary carcinoid tumor and neuroendocrine tumor of the lung.

**Fig. 1:**
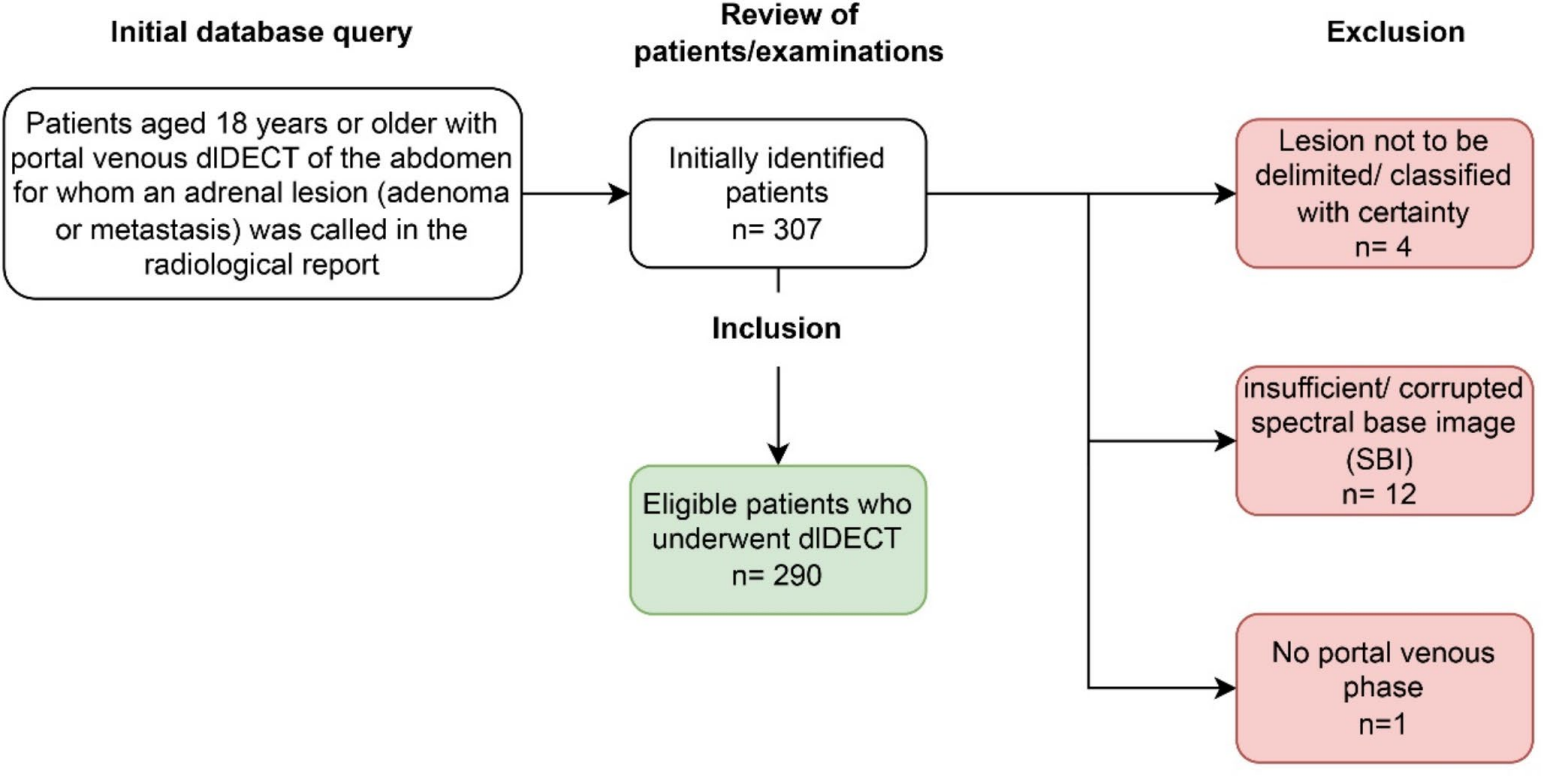
Overview on inclusion and exclusion of patients

Figure 1 provides a flowchart for inclusion and exclusion of study subjects.

### Reference standard and inclusion of lesions

Adrenal lesions were only included after they were unequivocally deemed metastatic or benign. The reference standards used to determine lesions as adenomas were (1) an unenhanced attenuation image attenuation of 10 HU or less, indicating microscopic fat, (2) consistency in size over at least 12 months without anti-cancer treatment, (3) unequivocal diagnosis as adenoma in dedicated adrenal imaging such as chemical-shift MRI or adrenal CT, and (4) histopathology.

For adrenal metastases, the reference standards were (1) interval new appearance of lesions, (2) increase in size in less than or equal to 6 months over 30%, (3) unequivocal diagnosis as metastasis in dedicated adrenal imaging such as chemical-shift MRI or adrenal CT, (4) histopathology. According to the reference standard, 169 adrenal metastases and 179 adenomas were deemed eligible for inclusion.

### Image acquisition and reconstruction

All patients underwent abdominal CT imaging on a dlDECT (iQon, Philips Healthcare, Best, The Netherlands). Every scan was performed in supine position. The following scan parameters were used: Tube voltage 120 kVp, tube current modulation with DoseRight index 17 (DoseRight 3D-DOM, Philips Healthcare), pitch 0.671, rotation time 0.33 s, collimation 64 × 0.625 mm, and matrix 512 × 512. A body weight-matched bolus of iodine-containing contrast agent followed by 30-mL saline was administered via antecubital vein (< 55 kg, 1 mL/kg; 55–120 kg, 100 mL; > 120 kg, 120 mL; Accupaque 350 mg/mL, GE Healthcare). Standard flow rate was set at 3.5 mL/s. PV images were acquired with a delay of 50 s after reaching the threshold of 150 HU in the descending aorta. Mean CT dose index (CTDI) was 11.3 ± 4.2 mGy. VUE and PV images were reconstructed with a slice thickness and section increment of 2 mm each in the axial plane using a hybrid iterative spectral reconstruction algorithm (Spectral, filter B, denoising level 3, Philips Healthcare).

### Image analysis

**Fig. 2:**
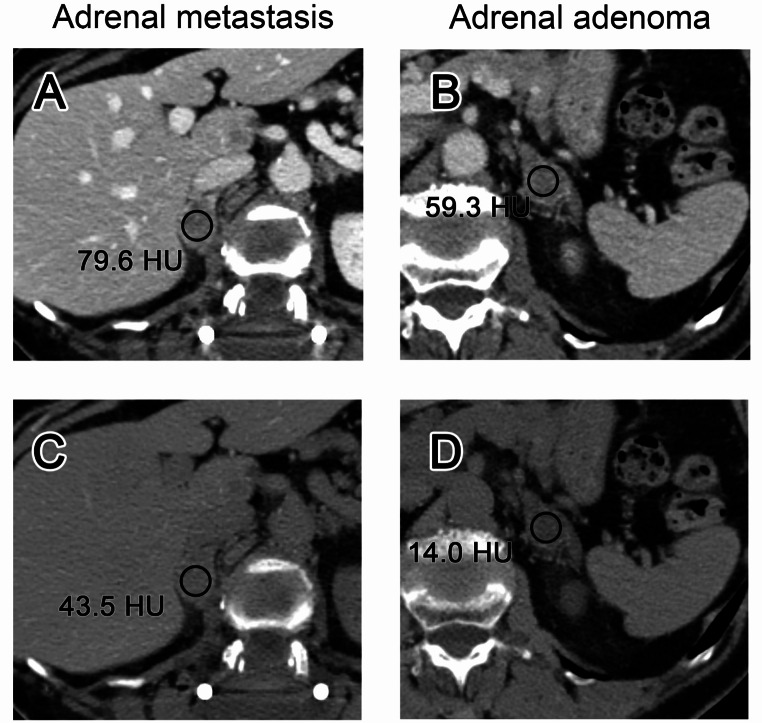
Examples of ROI placement in one adrenal metastasis and one adrenal adenoma. Portal venous phase (**a**) and virtual unenhanced (VUE) images (**c**) of a 75 year old woman with breast cancer and a right-sided adrenal metastasis and of a 75 year old woman with adenoma of the left adrenal gland (**b**, **d**), which was confirmed by true unenhanced images, with corresponding attenuation values

To obtain VUE attenuation, two unblinded readers placed circular regions of interest (ROIs) in adrenal lesions that were included in the analysis according to above-mentioned reference standards. All ROIs were placed on the contrast-enhanced images and afterwards automatically copied and pasted to the VUE images. The axial plane with the maximum lesion diameter was chosen, and the ROI was placed at the center of the corresponding lesion as to include a maximum amount of representative tissue while avoiding inclusion of unrepresentative or extra-adrenal voxels. Moreover, if present, inclusion of necrosis or degenerative changes was avoided. Figure 2 shows an example of ROI placement in one metastasis and one adenoma with corresponding VUE attenuation measurements.

### Validation of external VUE Attenuation thresholds for adrenal lesion characterization

A targeted literature research was performed aiming to identify studies that previously investigated dlDECT based adrenal lesion classification. Studies were identified using a combination of the search strings “((adrenal adenoma) OR (adrenal lesion)) AND (dual-energy ct)” and “((adrenal adenoma) OR (adrenal lesion)) AND (spectral ct)” in Pubmed. Studies investigating dual-layer dual-energy CT-derived virtual unenhanced images for diagnosing adrenal adenoma were identified from the list of results. Three previous studies in which quantitative VUE thresholds were reported were selected: Nagayama et al. reported a mean VUE threshold of 29 HU for adrenal adenoma diagnosis [12], Cao et al. reported a threshold of 22 HU [10] and Laukamp et al. [11] derived a threshold of 20 HU. In addition, a meta-analysis providing an aggregated sensitivity/specificity for the established 10 HU true unenhanced attenuation threshold was retrieved for reference [13]. The thresholds suggested in the studies were applied to our cohort to test the corresponding sensitivity, specificity and accuracy at identifying adrenal adenomas.

### Development of a new VUE Attenuation threshold for adrenal lesion characterization

In addition to testing previously reported thresholds, a receiver operating characteristic (ROC) analysis was performed to determine the optimal VUE threshold for differentiation between adrenal adenomas and metastases based on our patient cohort. The analysis included five-fold cross validation to avoid overfitting; here, the dataset was divided in five random, non-overlapping splits of 70 lesions, each. Since a total of 348 lesions was included, one split contained only 68 lesions; for reasons of simplicity, this split is considered to contain 70 lesions in the following analysis. Consecutively, five alternating training sets of 280 lesions were assembled from four splits each to perform the ROC analysis. The optimal VUE threshold of each training set was noted. Lastly, the obtained thresholds were tested on five non-overlapping test sets, to achieve the final model. In addition to the threshold yielding optimal accuracy according to ROC analysis and five-fold cross validation, a threshold for attaining a 98% specificity, which has been reported for the 10 HU unenhanced attenuation threshold [13] was calculated.

### Subgroup analysis of primary tumors on adrenal lesion characterization accuracy

To investigate the possible influence of underlying tumors in patients with adrenal metastases on differentiation from adenomas, the cohort was divided into four subgroups, considering the most frequent tumour entities in the patient group with adrenal metastases: (1) melanoma, (2) lung cancer, (3) renal cell carcinoma (RCC) and all other primary tumors not included in these groups, i.e., (4) others. For each of those subgroups, a dedicated ROC analysis including evaluation of sensitivity/specificity/accuracy for the corresponding thresholds was performed, as mentioned below.

### Statistical analysis

Statistical assessment was performed in *R* language for statistical computing, *R* Foundation, Vienna, Austria, version 4.0.0. Plots were created using the *R* library *ggplot2*, elegant graphics for data analysis [14]. ROC analysis was performed using the *R* library *pROC* [15]. Best thresholds of the ROC analysis were determined by Youden’s method. 95% confidence intervals were computed with 2000 stratified bootstrap replicates of the ROC data. Inter-reader variability was monitored using the intraclass correlation coefficient (ICC), and values < 0.5, 0.5–0.75, 0.75–0.9, and > 0.90 were considered to indicate poor, moderate, good, and excellent reliability, respectively [16].

## Results

### Quantitative analysis

Mean diameter of metastases was larger than of adenomas (18.3 ± 11.4 mm, interquartile range (IQR): 10.8–25.15 mm vs. 11.1 ± 4.7 mm, IQR: 8.27–13.5 mm; *p* < 0.001). Mean VUE attenuation in metastases was significantly higher than that in adenomas (30.9 ± 7.9 HU, IQR: 26.25–35.5 HU vs. 17.3 ± 16.0 HU, IQR: 11.75–24.42 HU; *p* < 0.001). The mean contrast enhanced attenuation in PV phase images showed a similar distribution, being significantly higher in metastases compared to adenomas (67.7 ± 23.0 HU, IQR: 51.8–82.5 HU vs. 56.0 ± 31.6 HU, IQR: 39.12–72.25 HU; *p* < 0.001). The ICC for the VUE measurements was excellent between both readers who performed the quantitative measurements (ICC = 0.93; 95% CI: 0.91–0.94).

### Validation of external thresholds for adrenal lesion characterization

**Fig. 3:**
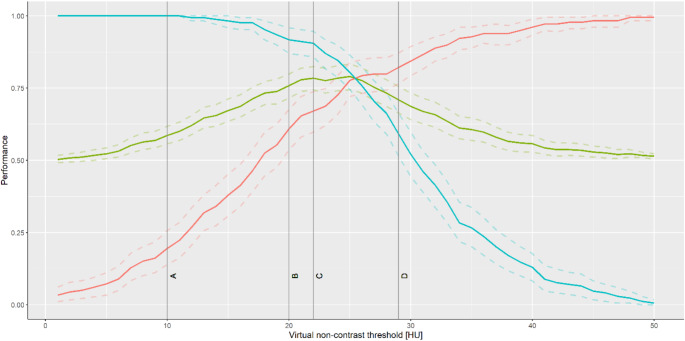
Validation of virtual non-contrast attenuation thresholds to discriminate adrenal adenomas from metastases. Line A: 10 HU true non-contrast attenuation threshold applied to VUE measurements. Line B: Laukamp et al.: 20 HU [11]. Line C: Cao et al.: 22 HU [10]. Line D: Nagayama et al.: 29 HU [12]. The red line represents sensitivity, the blue line specificity and the green line accuracy for diagnosis of adrenal adenoma, respectively, with the dotted lines representing the 95% confidence intervals

The mean VUE thresholds for adrenal adenoma diagnosis reported in the 3 selected previous studies by Nagayama et al., Cao et al. and Laukamp et al. and the corresponding respective sensitivity/specificity/accuracy when applied to this study’s cohort are shown in Table 1. Figure 3 shows the threshold-dependent performance of previous thresholds with 95% confidence intervals based on 2000 stratified bootstrap replicates of the ROC data.

**Table 1:** Sensitivity, specificity and accuracy for adrenal lesion differentiation based on thresholds from 3 previous studies including 95% confidence intervals with 2000 stratified bootstrap replicates of the ROC data

	Threshold (HU)	Sensitivity (95% CI)	Specificity (95% CI)	Accuracy (95% CI)
Cao et al. [10]	22	0.67 (0.6–0.74)	0.91 (0.86–0.95)	0.78 (0.74–0.82)
Nagayama et al. [12]	29	0.82 (0.77–0.88)	0.59 (0.51–0.66)	0.71 (0.66–0.76)
Laukamp et al. [11]	20	0.61 (0.54–0.68)	0.92 (0.88–0.96)	0.76 (0.72–0.80)

### Development of VUE Attenuation threshold

The results of each test split of the five-fold cross validation are shown in Table 2. The test results showed an accuracy range of 0.74 to 0.81. The final model yielded a mean threshold of 24.7 HU, and a mean sensitivity/specificity/accuracy of 0.76/0.81/0.79. For a 98% specificity, which had been reported for the established 10 HU unenhanced attenuation threshold, the corresponding VUE threshold was 17.3, yielding a sensitivity/specificity/accuracy of 0.49/0.98/0.73.

**Table 2:** Results of each training and test split of the five-fold cross-validation

	VUE Threshold (HU)	Sensitivity	Specificity	Accuracy
Train 1	23.7	0.73	0.89	0.8
Test 1	23.7	0.68	0.81	0.75
Train 2	24.9	0.76	0.84	0.80
Test 2	24.9	0.83	0.76	0.8
Train 3	25	0.77	0.80	0.78
Test 3	25	0.8	0.86	0.83
Train 4	24.9	0.79	0.84	0.81
Test 4	24.9	0.72	0.76	0.74
Train 5	24.9	0.78	0.81	0.79
Test 5	24.9	0.77	0.86	0.81
Mean (TEST)	24.7	0.76	0.81	0.79

The last line indicates the mean virtual unenhanced (VUE) attenuation threshold, sensitivity, specificity and accuracy attained, averaging the results of all 5 test splits

### Subgroup analysis of primary tumors on adrenal lesion characterization accuracy

In the subgroup of patients with adrenal metastases from melanoma, the mean threshold was 24.9 HU with a mean sensitivity/specificity/accuracy of 0.78/0.84/0.79. The corresponding threshold for lung cancer was 25.1 HU with a mean sensitivity/specificity/accuracy 0.78/0.8/0.78. For patients with metastases from RCC, the mean threshold was 25.1, with a mean sensitivity/specificity/accuracy of 0.78/1/0.79. For the group including various different primary tumors, the derived threshold wad 23.8 HU, with a sensitivity/specificity/accuracy of 0.73/0.85/0.76. Results of the subgroup analysis are summarized in Table 3.

**Table 3:** Sensitivity, specificity and accuracy of thresholds derived from different subgroups of patients with metastases regarding differentiation of these lesions from adrenal adenomas

Tumor	Threshold (HU)	Sensitivity	specificity	Accuracy
Melanoma	24.9	0.78	0.84	0.79
Lung Cancer	25.1	0.78	0.8	0.78
Renal Cell Carcinoma	25.1	0.78	1	0.79
Others	23.8	0.73	0.85	0.76

## Discussion

This study evaluated previously suggested VUE attenuation thresholds for dual-layer dual-energy CT (dlDECT)-based differentiation of adrenal lesions in a large retrospective cohort. Thresholds from three different studies were retrieved [10–12], which resulted in a varying respective accuracy when being applied to our cohort. Moreover, the respective sensitivity and specificity of the individual thresholds that resulted from the testing of before-mentioned thresholds in our cohort diverged from those reported in the corresponding manuscripts, which can be explained by the fact that the models were overfitted to the training data. For example, whereas Cao et al. reported a sensitivity/specificity of 0.82/0.85 for the 22 HU cutoff [10], this cutoff led to a markedly lower sensitivity of 0.67, but a higher specificity of 0.91 when applied to our study cohort. The cross-validated threshold derived from our cohort was 24.7 HU, resulting in an accuracy at adrenal lesion differentiation of 0.79.

For the diagnosis of adrenal adenoma, a high specificity is of utmost importance in order to avoid calling false positives, i.e., to misdiagnose metastatic lesions, which are the most likely differential diagnoses in the setting of oncologic imaging, as adenomas. In this context, we found that for a specificity of 0.98, which has been reported for the 10 HU unenhanced attenuation threshold, a VUE threshold of 17.3 HU could be used. Notably, the corresponding sensitivity for that specificity-focused threshold was poor (0.49) compared to the sensitivity of the 10 HU unenhanced attenuation threshold, which Boland et al. reported to be 0.71 in a meta-analysis including different studies on CT-based adrenal lesion differentiation [13]. The underlying reason for this reduced sensitivity at a given high specificity is the overestimation of adrenal attenuation in VUE images that has been reported in various studies [10, 12, 17, 18]. Adenomas typically contain intracytoplasmic fat along with soft tissue elements and exhibit iodine enhancement. One possible reason for the overestimation is that the two-material decomposition algorithm used in dlDECT may lose accuracy when a third material is present. Consequently, the optimized threshold yielding the best accuracy, i.e., the most balanced tradeoff between sensitivity and specificity, was higher than the traditional 10 HU, which is in line with all previous studies on dlDECT-based adrenal lesion differentiation that were analyzed. For best clinical practice, the 17.3 HU threshold might be more feasible than the one with the best overall accuracy, due to the importance of avoiding false positive diagnoses of adenomas in the incidental setting, i.e., missing metastatic lesions that could alter treatment and prognosis.

An important aim of our study was to investigate whether the underlying tumor in patients with adrenal metastases impacts the diagnostic accuracy with which those can be distinguished from adrenal adenomas. This has not been elucidated before, probably due to sample size restrictions of previous studies. In our study, patients with adrenal metastases were divided into four major subgroups. It was found that the thresholds derived from those subgroups showed only negligible variations (range: 23.8–25.1 HU). This is an important finding as it underscores the generalizability of the proposed VUE threshold across patients with various malignant diseases.

Given the overestimation of true unenhanced attenuation in virtual unenhanced images, alternative means to attain high accuracy of differentiation have been proposed. It is known that both a rapid wash-in as well as a washout of contrast media are imaging features suggestive for adenoma [19, 20]. Nagayama et al. described that a ratio of iodine concentration and VUE attenuation was superior to VUE attenuation alone in differentiating adrenal adenomas from metastases [12]. We did not include this approach in our study, as we aimed to investigate the best possible performance of the monoparametric approach based on VUE, which is more easily applicable in daily clinical routine. However, testing this promising approach in a larger cohort should be subject to further investigations.

Our study has further limitations that need to be addressed. Besides the monocentric and retrospective design, we did only include one particular dual-energy CT scanner, the detector-based dlDECT. Although the overestimation of VUE values has been described for other DECT scanners as well, our findings cannot be generalized to other DECT systems, particularly in view of the known inter-scanner differences of DECT-derived VUE attenuation [21, 22]. Due to the fact that an unenhanced acquisition is not performed at baseline as standard at our institution, comparing VUE and true unenhanced attenuation could not be performed to further solidify previous results on quantitative VUE accuracy. Moreover, our ground truth did not allow to distinguish between lipid-poor and lipid-rich adenomas, as chemical shift imaging and/or dedicated adrenal CT protocols were not available for many of the patients included, which however can be considered of limited practical relevance in the clinical scenario of an incidentaloma that needs to be classified in a monophasic examination.

To conclude, previous DECT-derived VUE thresholds vary in terms of their diagnostic accuracy for differentiating adenomas and metastases. The cross-validated VUE threshold of 24.7 HU yielded an accuracy of 0.79, whereas a threshold of 17.3 HU was found to provide comparably high specificity as reported for the 10 HU unenhanced attenuation threshold, albeit with a reduction in sensitivity.

## Data Availability

The datasets used and/or analyzed during the current study are available from the corresponding author on reasonable request.

## Abbreviations

DECT: Dual-energy CT
dlDECT: Dual-layer detector dual-energy CT
HU: Hounsfield Units
PACS: Picture archiving and communication system
PV: Portal venous
RIS: Radiological information system
SBI: Spectral base image
VUE: Virtual unenhanced attenuation
